# Regulation of ethyl ester synthesis in two apple (*Malus domestica*) cultivars: Insights from integrated metabolomic and transcriptomic analyses

**DOI:** 10.1016/j.fochms.2025.100282

**Published:** 2025-08-05

**Authors:** Fukuyo Tanaka, Ryoichi Yano, Keiki Okazaki, Hikari Kuchikata, Jimpachi Masuda, Satoshi Kasai, Miho Tatsuki

**Affiliations:** a National Agriculture and Food Research Organization (NARO), Research Center for Advanced Analysis, 3-1-3 Kannondai, Tsukuba, Ibaraki 305-8604, Japan; b Research Center for Advanced Analysis, NARO, 2-1-2 Kannondai, Tsukuba, Ibaraki 305-8518, Japan; c Institute for Agro-Environmental Sciences, NARO, 3-1-3 Kannondai, Tsukuba, Ibaraki 305-8604, Japan; d Apple Research Institute, Aomori Prefectural Industrial Technology Research Center, 24 Fukutami, Botandaira, Kuroishi, Aomori 036-0332, Japan; e Institute of Fruit Tree and Tea Science, NARO, 2-1 Fujimoto, Tsukuba, Ibaraki 305-8605, Japan

**Keywords:** 1-MCP treatment, Acetate esters, Ethanol pathway, Ethylene production, ‘Orin,’ PDC function, ‘Shinano gold’

## Abstract

Although ethyl esters are known to accumulate under hypoxic conditions, their biosynthetic regulation during senescence under aerobic conditions remains unclear. We hypothesized that ethylene could play a pivotal role in the ethyl ester synthesis under aerobic conditions. We conducted an integrated analysis of metabolite and transcript profiles over time using ‘Orin’ and ‘Shinano Gold’ apples, both with and without 1-MCP treatment. Comparative analysis of the ethanol pathway and ethyl ester-related genes revealed that two PDC genes, *MdPDC2* and MD04G1159900, are rate-limiting for ethyl ester synthesis in ‘Orin’, in proportion to ethylene production. In contrast, ethanol and ethyl ester concentrations in ‘Shinano Gold’ were lower than expected based on ethylene production, suggesting that PDC and/or ADH function in the ethanol synthesis may be impaired. These regulatory mechanisms governing ethyl ester synthesis during aerobic ripening provide valuable insights for optimizing storage strategies and for breeding cultivars with enhanced aroma profiles.

## Highlights

• Ethylene promoted the synthesis of ethyl esters via ethanol under aerobic conditions.
• Ethyl ester concentrations in ORN were strongly correlated with ethylene production.
• Expression of *MdPDC2* and/or MD04G1159900 was vital for ethyl ester synthesis.
• Low concentrations of SG ethyl esters were linked to impaired PDC and/or ADH function.
• Ethanol availability, not AAT activity, limited ethyl ester synthesis.

## Graphical abstract

**Figure:**
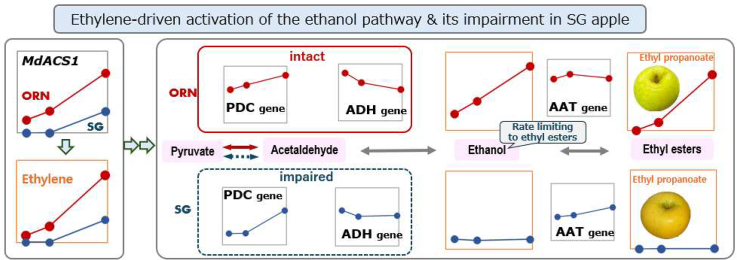

## 1 Introduction

Flavor is an important determinant of apple preference, just like texture, with aroma compounds playing a crucial role. Many apple cultivars exhibit characteristic aromas composed of lipid-degrading metabolites such as esters, alcohols, aldehydes, and fatty acids, along with terpenoids, phenylpropenes, furans, lactones, and sulfur-containing compounds (Fuhrmann & Grosch, 2002; Li, Yan, et al., 2023; Soomro et al., 2023; Yang et al., 2023). In Japan in particular, flavorful apple varieties such as ‘Fuji,’ ‘Orin,’ and ‘Jonagold,’ which have high ester content, are predominant (Igarashi et al., 2016). The ester composition is a key factor in defining flavor characteristics and preferences. Among esters, acetate esters—formed by the condensation of various alcohols with acetic acid—are known for their fruity aroma and green freshness, making them highly desirable aromatic components (Aprea et al., 2017). Acetate esters are also abundant in several fruits, including pears, peaches, strawberries, bananas, melons, and pineapples, and are similarly described as conveying a fruity aroma in each (Asikin et al., 2022; Komthong et al., 2006; Li et al., 2016; Li, Wang, et al., 2023; Mehinagic et al., 2006; Ueda et al., 1992). Notably, acetate esters of C6 alcohols are especially abundant in Rosaceae fruits; for example, hexyl acetate is a key aroma volatile in European pears, apples, and strawberries (Abouelenein et al., 2023; Holland et al., 2005; Liu et al., 2025; Rowan et al., 2009). Highly volatile aromatic components such as ethyl esters also possess a fruity-sweet aroma and typically have a low olfactory threshold (Cliff et al., 2011; Niu et al., 2019; Van Gemert, 2003). For this reason, ethyl esters are considered to be closely linked to human palatability. For instance, watercored apples are highly favored by consumers in Japan, China, and other Asian countries, due to their mellow sweet aroma enriched with complex ethyl esters (Tanaka et al., 2016; Tanaka et al., 2020). The exposure of apple fruit to gaseous ethanol led to an increase in ethyl ester content, which was subsequently evaluated as having a strong sweet apple-like aroma in sensory assessments (Tanaka et al., 2020). In addition to apples, ethyl esters are also found in various fruits, including bananas, pineapples, and strawberries, as well as in fermented foods and beverages such as wine, Japanese sake, and various distilled alcohols (Asikin et al., 2022; Liu et al., 2023; Mimura et al., 2014; Munoz-Gonzalez et al., 2019; Shi et al., 2018; Styger et al., 2011; Tokitomo et al., 2005; Zhao et al., 2020). For example, two ethyl esters—ethyl 2-methylpropanoate and ethyl 2-methylbutanoate—have been identified as key odorants responsible for the fruity aroma in fresh pineapple (Tokitomo et al., 2005). Ethyl hexanoate is known as a major component of the scent of *ginjo-sake,* one of the most popular types of Japanese sake, presenting an apple-like sweet aroma and contributing to the palatability of *ginjo-sake* (Kanno et al., 2021; Mimura et al., 2014). On the other hand, ethyl esters are also associated with the smell of fermentation, senescence, or decay in food materials (Zhao et al., 2024). In apples, ethyl esters are produced after long-term cold air storage, depending on the cultivar, during a process known as senescence (Argenta et al., 2004; Li, Yan, et al., 2023). Whether ethyl esters are perceived as desirable depends on their concentration and balance with other volatile and soluble components. Understanding the amount and balance of ethyl esters in apples and the mechanisms regulating them is therefore crucial to determining their palatability.

It is widely accepted that ethanol, the substrate for ethyl esters, is produced through ethanol fermentation under low-oxygen conditions. In apples, the accumulation of ethanol and ethyl esters has been observed when the flesh is exposed to low-oxygen environments, such as controlled atmosphere (CA) storage or in watercored apples (Dixon & Hewett Errol, 2000). This process redirects the glycolytic metabolic flow to the ethanol pathway under hypoxic conditions via the action of pyruvate decarboxylase (PDC), which converts pyruvate to acetaldehyde. As Li et al. (2022) reported, the expression of two PDC genes, *MdPDC1* (MD10G1238500) and *MdPDC2* (MD4G1160100), was observed to be upregulated when apples were subjected to hypoxic conditions. Furthermore, Wada et al. (2021) also demonstrated that *MdPDC1* was upregulated in the watercored flesh tissue compared to unaffected flesh tissue from the same watercored apple. These findings suggest that these PDC genes play a significant role in ethanol and ethyl ester synthesis under hypoxic conditions. However, since fruit ripening or senescence does not always coincide with hypoxia, the production of ethanol-related metabolites under aerobic conditions cannot be solely attributed to maintaining cellular redox balance (Boeckx et al., 2019).

It is postulated that distinct regulatory mechanisms are involved in the synthesis of ethyl esters under low-oxygen conditions and during ripening or senescence. In this study, these mechanisms are referred to as hypoxia-driven and ethylene- or ripening/senescence-driven activation of the ethanol pathway, respectively. Notably, while acetate esters are prevalent in many apple cultivars, the concentration of ethyl esters varies significantly depending on the cultivar and environmental conditions, such as cultivation method, harvest time, and storage period and conditions (Fellman et al., 2003; Horiuchi et al., 1986; Roberts & Spadafora, 2020; Young et al., 2004). In light of these considerations, the objective of the experiment was to elucidate the differences between low-oxygen conditions and the regulation of ethyl ester formation during ripening or senescence and to identify the underlying mechanisms responsible for the observed variation in ethyl ester production between cultivars, with a particular focus on gene expression in the ethanol pathway.

Ethylene is well established as a stimulator of ester biosynthesis (Defilippi et al., 2005; Fan et al., 1998). The strong ethylene-action inhibitor, 1-methylcyclopropene (1-MCP), binds more effectively to ethylene receptors than ethylene itself, thereby physiological action of ethylene (Blankenship & Dole, 2003; Tatsuki et al., 2007; Watkins et al., 2000). Prior studies demonstrated that 1-MCP treatment in ‘Golden Delicious’ significantly reduced ethylene production and suppressed the formation of several esters, including hexyl acetate, 2-methylbutyl acetate, and butyl butanoate (Yang et al., 2013; Yang et al., 2016). Ethylene production varies considerably among cultivars (Kikuchi et al., 2017; Wang et al., 2009), suggesting that each may exhibit a unique ester profile (Fan et al., 1998; Fan & Mattheis, 2001). However, the precise influence of ethylene on ethyl ester synthesis is not yet fully elucidated. Thus, further studies are required to clarify the involvement of ethylene and its related genes in regulating the synthesis of ethyl and acetate esters, particularly in a cultivar-specific manner. Gaining insights into these molecular mechanisms is vital for improving storage strategies and for breeding cultivars with enhanced aroma profiles.

The experiment was conducted using two Japanese-bred cultivars: ‘Orin’ (ORN) and ‘Shinano Gold’ (SG), also known as Yello in Europe. These cultivars are among the most popular in Japan (Igarashi et al., 2016) and are known for their distinctive aroma characteristics. The cultivars were evaluated with and without 1-MCP treatment to elucidate the role of ethylene.

## 2 Materials & methods

### 2.1 Plant materials

An integrated metabolomic and transcriptomic study was conducted on two apple cultivars: SG (‘Golden Delicious’ × ‘Sensyu’ [‘Toko’ × ‘Fuji’]) and ORN (‘Golden Delicious’ × ‘Indo’) using fruits cultivated in 2020 at the Apple Research Institute of the Aomori Prefectural Industrial Technology Research Centre (Aomori, Japan). The fruits were harvested at their respective maturity dates—25 October for SG and 1 November for ORN—and stored at 0 °C until transport to the National Agriculture Research Organization (NARO) in Ibaraki Prefecture on 4 November. Upon arrival the following day, the ORN fruits were returned to 0 °C storage, while the SG fruits underwent 1-MCP treatment on the same day.

**Fig. 1:**
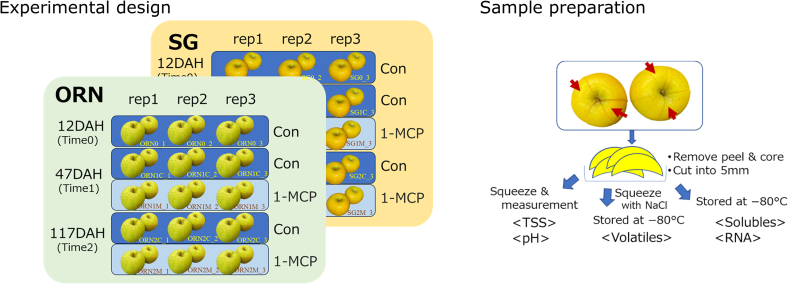
Experimental design and sample preparation.

Treatment with 1-MCP was performed 10 days after harvest (DAH), following the method described by Tatsuki et al. (2007). This occurred on December 5 and 11 for SG and ORN, respectively. The fruits were placed in 56-L plastic containers and exposed to 1 ppm 1-MCP (SmartFresh, AgroFresh Inc., Springhouse, PA, USA) for 16 h at 22 °C. The control group, which did not receive 1-MCP treatment, was kept in air under the same conditions. Following treatment, all fruits were stored at 0 °C. At the initial sampling (Time 0), only fruits from the control group were used, 12 DAH. The second and third samplings, Time 1 and Time 2, conducted at 43 and 117 DAH, respectively, included both control (CON) and 1-MCP-treated (MCP) fruits. Accordingly, the sample codes were set as SG0, SG1C, SG2C, SG1M, and SG2M for SG and ORN0, ORN1C, ORN2C, ORN1M, and ORN2M for ORN (Fig. 1). Fruits were removed from 0 °C storage 16 h prior to ethylene measurement and maintained at 20 °C. Sampling times were defined as follows: Time 0 represented the harvest point (prior to the ethylene burst during cold storage). Time 1 indicated the stage at which the effects of 1-MCP treatment were expected to become evident, while Time 2 corresponded to the stage just before visible signs of physiological disorder due to senescence in untreated ORN fruit.

### 2.2 Sample preparation for fruit quality, metabolites, and gene expression analysis

Six fruits used per treatment were divided into three biological replicates of two fruits each (Fig. 1). After measuring ethylene production, fruit weight, and firmness (below), each fruit was sectioned vertically into six parts, and the peel was removed. The flesh from two opposite sections of each fruit was combined to form one biological replicate and cut into 5 mm pieces. Twenty grams of the cut flesh were immediately frozen in liquid nitrogen and stored at −80 °C for subsequent RNA extraction and analysis of water-soluble components. For total soluble solid (TSS) and pH measurement, 25 g of the cut flesh were mashed using a food processor and squeezed through double gauze to extract juice. For volatile analysis, juice was prepared similarly using 40–50 g of flesh mixed with 20 % sodium chloride (FUJIFILM Wako Pure Chemical Corporation, Osaka, Japan), portioned into 1.5-mL microtubes (1.3 mL per tube), and stored at −80 °C until analysis.

### 2.3 Fruit general quality

Ethylene production was measured using a gas chromatograph (GC) equipped with flame ionization detectors (Shimadzu GC-2014, SHIMADZU CORPORATION, Kyoto, Japan) and a PoraPlot N column, under analytical conditions previously described (Tanaka et al., 2015). Individual fruits were placed in 1.2-L airtight glass chambers at 20 °C for 1 h. One milliliter of headspace gas was withdrawn and injected into the GC system. Firmness was assessed on opposite sides of each fruit using a penetrometer (FT327, 15-mm diameter, ITALTEST, Milano, Italy) and expressed in newtons (N). TSS and pH were measured from the prepared juice using a PR-101a refractometer (ATAGO CO., LTD., Tokyo, Japan) and a LAQUA twin pH meter (HORIBA, Ltd., Kyoto, Japan), respectively.

### 2.4 Volatiles and aqueous metabolites analysis

Analysis of volatiles was carried out according to Honda et al. (2024) with minor modifications. In brief, a trap material (MonoTrap™, custom-made without holes inside the rod of RGPS TD; GL Science Co. Ltd., Tokyo, Japan) was used to extract volatile components from the juice. For instrumentation, a thermal desorption (TD) unit (TDU; GERSTEL GmbH & Co. KG, Mülheim an der Ruhr, Germany), a programmable temperature vaporization system (CIS4, GERSTEL), and an Agilent 5977B mass spectrometer with a 7890B gas chromatograph (Agilent Technologies Inc., Palo Alto, CA, USA) were used as the TD-GC–MS system. A DB-HeavyWax analytical column (length: 60 m, inner diameter: 0.25 mm, film thickness: 0.25 μm; Agilent Technologies) with an inactivated fused silica tube (0.57 m in length, 0.10 mm inner diameter) at the end was employed. The instrument settings were the same as those reported in Honda et al. (2024). Data analysis was performed using MassHunter Unknowns Analysis (ver. B.09.00, Agilent Technologies) in combination with Aroma Office 2D software ver. 7.0 (Gerstel KK, Tokyo, Japan). Mass spectral searches were conducted using the NIST20 and in-house libraries with a minimum MS match score threshold of 80. Retention index matches were verified using the in-house library specific to the DB-HeavyWax column, the Agilent off-flavor database, and the Aroma Office 2D database specific to the DB-Wax column, with an allowable retention index deviation of ±20. The target list, including component retention times and quantifier ions (Table S1), was imported into MassHunter Quantitative Analysis software for peak integration.

For water-soluble metabolite analysis, approximately 20 g of frozen apple flesh was used after freeze-drying and powdering. Metabolites were extracted using a methanol–chloroform–water solution (3:1:1) at 20 °C for 10 min. The supernatant was then freeze-dried under vacuum and further dehydrated overnight in a desiccator. The extract was supplemented with ^13^C_6_-sorbitol (CIL, Tewkesbury, MA, USA) as an internal standard. Samples were loaded into the instrument following derivatization with methoxyamine hydrochloride (Sigma-Aldrich, St. Louis, MO, USA) and MSTFA (*N*-methyl-*N*-trimethylsilyltrifluoroacetamide, Thermo Fisher Scientific, Waltham, MA, USA). A GC–MS system (Shimadzu GC–MS-2010QP Ultra, SHIMADZU CORPORATION) equipped with an Rtx-5Sil MS column with an integrated guard column (30 m, 0.25 mm film thickness; Restek GmbH, Bad Homburg, Germany) was used for analysis. Metabolite annotation was performed using MS spectra and retention index values from a user-defined library as described Okazaki et al. (2020). Area values of target solubles were accumulated using MassHunter Quantitative Analysis software and standardized relative to the sorbitol internal standard.

### 2.5 Statistical analysis of metabolites

For metabolite data processing, raw peak area values and standardized area values relative to internal standards were used for volatiles, including ethylene, and aqueous metabolites, respectively. Principal Component Analysis (PCA) was performed using JMP software (ver. 13) after variables were mean-centered and scaled by their standard deviation to ensure equal variance. Additionally, significance tests were conducted to compare cultivars, 1-MCP treatment status, and sampling stages for TSS, firmness, pH, and ethylene concentration.

### 2.6 RNA isolation, RNA-seq data acquisition, and co-expression analyses

Total RNA was extracted from frozen samples using the hot borate method (Wan & Wilkins, 1994), following the protocol described by Wada et al. (2021). DNase I treatment (TURBO DNA-free Kit, Ambion, Austin, CA, USA) was applied to minimize DNA contamination. First-strand complementary DNA (cDNA) synthesis was performed using a High-Capacity cDNA Reverse Transcription Kit (Applied Biosystems, Foster City, CA, USA).

Libraries for 150-bp paired-end sequencing were prepared using the Illumina TruSeq Stranded mRNA Sample Preparation Kit and sequenced on an Illumina NovaSeq 6000 system through the outsourcing services of GENEWIZ Japan (Saitama, Japan). At least 20 M read-pairs were generated as raw data for each sample. The raw sequencing data have been deposited in the NCBI Sequence Read Archive under accession number PRJNA1255739 (https://www.ncbi.nlm.nih.gov/bioproject/?term=PRJNA1255739).

Illumina RNA-seq paired-end short reads were processed using Trimmomatic v0.36 (Bolger et al., 2014) to remove low-quality data, with the following parameters: ILLUMINACLIP: TruSeq3-PE-2.fa:2:30:10, LEADING:15, TRAILING:15, SLIDINGWINDOW:4:15, MINLEN:30. Clean reads were mapped to the GDDH13 v1.1 genome using HISAT2 v2.1.0 (Kim et al., 2019) with the following parameters: “-k 1 --maxins 1000 --score-min L,0,−0.12 --mp 2,2 --np 1 --rdg 1,1 --rfg 1,1 --min-intronlen 20 --max-intronlen 10000” for the first RNA-seq and “--min-intronlen 20 --max-intronlen 10000” for the second RNA-seq, respectively. Gene expression levels were quantified as transcripts per million (TPM) using StringTie (Pertea et al., 2015). Upper quantile normalization was applied to log2-transformed TPM values. Weighted genome-wide co-expression network analysis (WGCNA) (Langfelder & Horvath, 2008) was conducted to generate the co-expression dataset, following previously described methods (Yano et al., 2020; Yano et al., 2025). Prior to WGCNA, expressed gene and detectable volatiles were selected based on the following criterion: a TPM or volatile intensity >0.1 in at least six of the 30 samples (Table S4; Yano et al., 2020; Yano et al., 2025). A power threshold of 14 was applied for all analyses. In WGCNA, artificial numeric vectors (eigengenes) representing the expression profiles of each co-expression group (module) were automatically generated, along with *p* -values indicating the probability of module membership for each gene. Pearson's correlation coefficients were also calculated using R (v3.6.3) (R Core Team, 2021) between each gene's TPM values and the corresponding eigengene vectors. Genes were assigned to co-expression modules if the *p*-value was <1 × 10^−6^ and the module contained at least 10 members existed. Line charts were generated using R software.

### 2.7 InterProScan search and ID enrichment analysis

An InterProScan search was conducted to obtain GO terms and InterPro IDs for all protein-coding genes using the deduced protein sequence data from the apple genome reference GDDH13 v1.1 (Daccord et al., 2017). Term enrichment analysis (TEA) was performed based on this information using a two-tailed Fisher's exact test implemented in R (v3.6.3) with the exact2x2 package. The q-value package in R was used to calculate *q*-values from the corresponding *p-*values.

### 2.8 Search for putative apple homologs for arabidopsis

To identify apple homologs of Arabidopsis genes related to PDC, alcohol dehydrogenase (ADH), alcohol acyl transferase (AAT), and phytohormones, a BLASTp search was performed using the deduced protein sequences from the GDDH13 v1.1 apple genome reference as queries and the complete protein sequence dataset of TAIR (The Arabidopsis Information Resource, Arabidopsis Genome Initiative, 2000) as the database.

## 3 Results

### 3.1 Time course of fruit firmness, TSS, pH, and ethylene production

**Fig. 2:**
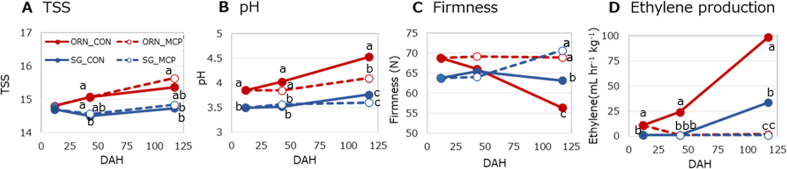
TSS, pH, firmness, and ethylene production. Values with different letters differ significantly at the 5 % level within each DAH; Turkey's HSD test.

At the first sampling conducted at 12 DAH (Time 0), the ORN cultivar exhibited a higher pH compared to SG, although no significant differences in TSS and firmness were observed. Ethylene was detected at 10.7 μL hr^−1^ kg^−1^ in ORN, but remained below 1 μL hr^−1^ kg^−1^ in SG (Fig. 2D). Complete suppression of ethylene production in 1-MCP-treated ORN was subsequently observed at 43 DAH. At 117 DAH, ethylene levels reached 98.3 μL hr^−1^ kg^−1^ in ORN and 33.4 μL hr^−1^ kg^−1^ in SG without 1-MCP treatment. However, 1-MCP treatment led to suppressed ethylene levels in both cultivars. The ORN cultivar showed a significant decrease in firmness and an increase in pH, while SG demonstrated comparatively smaller changes. It can be posited that the characteristics of firmness, TSS, and pH are influenced by ethylene production levels.

### 3.2 Flavor profiling over time and cultivar characteristics

The aroma compounds (130) and water-soluble components (23) analyzed in this study, along with their peak intensities, are listed in Table S1 and S2. The largest group of aroma compounds was esters, followed by alcohols, aldehydes, ketones (including lactones), and terpenes. Among the water-soluble compounds, four amino acids, nine fatty acids and organic acids, and ten sugars and sugar alcohols were also detected.

**Fig. 3:**
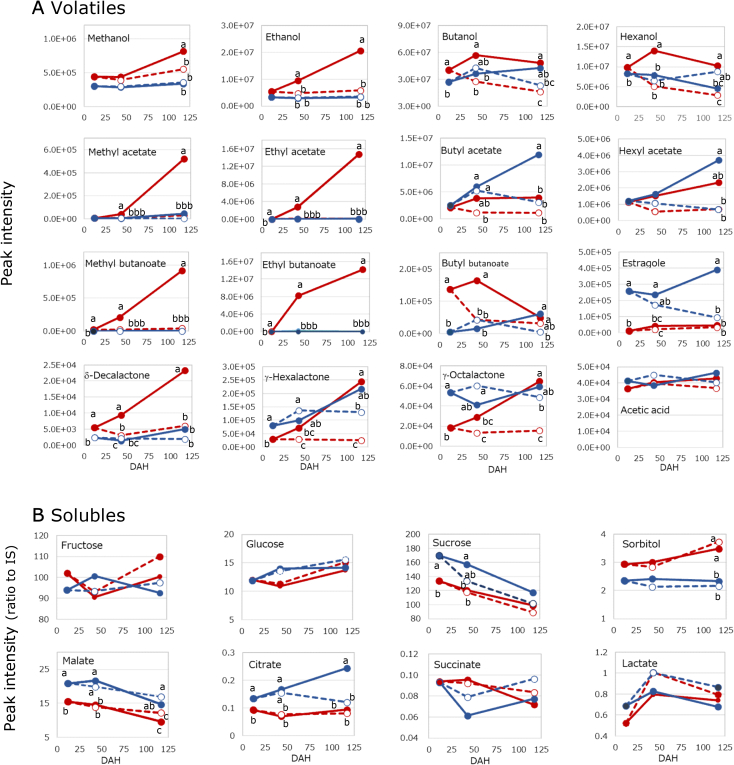
Time course of metabolites. A: volatile peak intensities. B: Soluble components relative to internal standard. Symbols are the same as in Fig. 2. Values with different letters differ significantly at the 5 % level within each DAH; Turkey's HSD test.

The time courses of major target volatiles such as ethyl and acetyl esters and alcohols and key solubles, including sugars and amino acids-are shown in Fig. 3. In ORN, the levels of methyl esters, ethyl esters, methanol, and ethanol exhibited strong concordance. These patterns were also closely aligned with ethylene trends shown in Fig. 2D. In SG, methanol and ethanol remained consistently low throughout the storage period. This suggests that the ethylene-dependent increase in ethanol and methanol may be suppressed in SG. Acetate esters were more abundant in SG than in ORN. The behaviors of methyl and ethyl acetates were more similar to those of ethyl esters than to acetates formed from other alcohols. γ-Lactones may also be characteristic of SG.

**Fig. 4:**
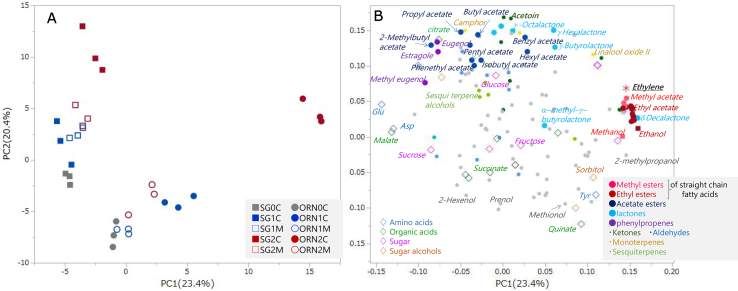
Principal component analysis (PCA) score plots (A) and loading plots (B) for volatile and soluble metabolites.

A PCA was performed to obtain an overview of the metabolite profiles of two apple cultivars treated with or without 1-MCP. Data for water-soluble and aroma components (Table S2), along with ethylene production, were combined and subjected to PCA. The score plot and loading plot for principal component 1 (PC1) and principal component 2 (PC2) are shown in Fig. 4A, B. The score plots indicate that both PC1 and PC2 were influenced by ethylene status, ripening stages, and cultivar differences. More specifically, the alteration along the PC1 axis was found to be reduced by 1-MCP treatment, particularly in ORN, suggesting that PC1 reflects the degree of ethylene-driven ripening. The cultivars were clearly separated along the PC2 axis. The PCA score plots visualized two key phenomena: (1) a substantial change in aroma components over time and (2) suppression of this change by 1-MCP treatment, indicating ethylene involvement (Fig. 4A, B). In contrast, although time-dependent changes in aroma components were observed in SG, the fluctuations were less pronounced than those in ORN. Additionally, the impact of 1-MCP treatment was smaller in SG compared to ORN, presumably due to SG's lower ethylene production.

The components with high loading in PC1 include ethanol, δ-decalactone, ethyl pentanoate, ethyl 3-(methylthio) propanoate, ethyl hexanoate, methyl hexanoate, ethyl acetate, and others (Fig. 4B; Table S3). A total of 16 of the top 20 components are methyl and ethyl esters or their substrates, ethanol. Ethylene was also included among these 20 components. Conversely, water-soluble components such as Glu, Asp, malate, and sucrose, along with phenylpropenes including estragole and methyl eugenol, exhibited low loading scores for PC1. These components are presumed to be potentially ethylene-insensitive or, alternatively, ethylene-inhibited. For PC2, high-loading components included 2-heptanone, acetoin, γ-nonalactone, γ-hexalactone, camphor, γ-octalactone, propyl acetate, δ-pentalactone, linalool oxide I, *p*-anisaldehyde, and butyl acetate. Water-soluble citrate was also included. The top 20 components in PC2 represented a diverse range, including five acetate esters, three ketones, four lactones, and two monoterpenes, distinct from PC1, which was dominated by contributions from methyl and ethyl esters. In contrast, the negative direction of PC2 reflected contributions from quinate, Tyr, low-molecular-weight fatty acids, aliphatic alcohols, and sugar alcohols. Ethylene, methyl esters, ethyl esters, methanol, and ethanol were closely positioned and associated with ORN and ripening/senescence. Straight-chain acetate esters and γ-lactones were found to cluster in a region characterized by high PC2 scores. In Fig. 4B, methyl acetate and ethyl acetate exhibited greater similarity to the methyl and ethyl esters shown in red than to the acetate esters shown in dark blue, consistent with Fig. 3.

### 3.3 Integrative correlation analysis of metabolites and transcriptome

#### 3.3.1 Overview of the WGCNA

A total of 30 transcriptome and metabolite intensity samples were combined and subjected to WGCNA. The dataset comprised 10 classes based on two cultivars, three time points, and 1-MCP-treated or untreated conditions. Of the 52,741 genes recorded in GDDH13 v1.1, 37,098 genes were annotated with GO/IPR. In this study, 31,502 genes were selected as transcribed genes, and 25,169 were grouped into 393 distinct modules using a power threshold of 14 (Table S4). The number of genes with normalized TPM values of 0.1 or higher was 28,127 for ORN and 27,557 for SG, corresponding to 53.3 % and 52.2 % of all genes in GDDH13 v1.1, respectively. Given the compatible gene expression coverage between the two cultivars, we conclude that GDDH13 v1.1 serves as a suitable reference genome for both.

**Fig. 5:**
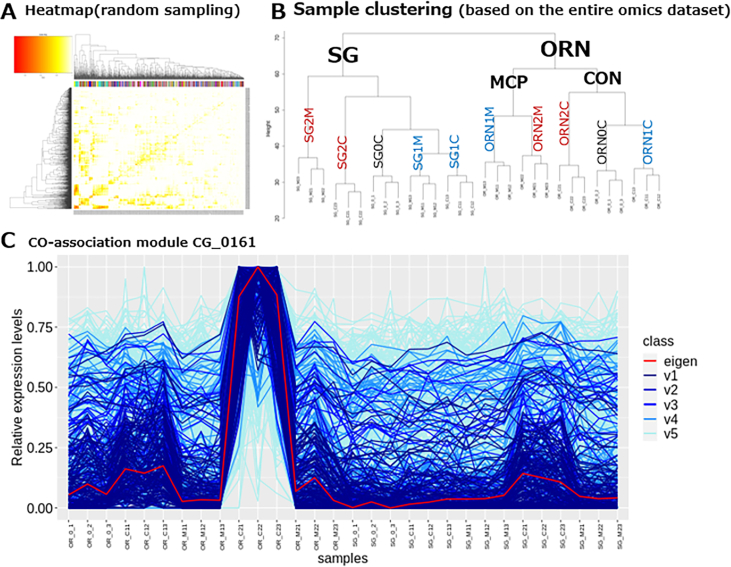
Co-expression groups identified by WGCNA in ORN and SG apples with and without 1-MCP treatment. (A) A heat map showing WGCNA clustering of apple metabolites and genes. (B) A phylogenetic tree showing eigengene relationships. (C) Co-association module including ethylene.

Fig. 5B presents an overview via a hierarchical clustering dendrogram. Overall, all biological replicates were distinct and clearly reflected differences between cultivars, 1-MCP treatment, and sampling stages. Samples were first grouped by cultivars. For ORN, samples were further separated by 1-MCP treatment and then by sampling stage. In contrast, for SG, the sampling stage preceded 1-MCP treatment in the clustering hierarchy. This suggests that the impact of 1-MCP was less significant in SG, likely due to its comparatively lower ethylene production (Fig. 2D).

#### 3.3.2 Ethylene-dependent regulation of gene expression and metabolite accumulation

Among the 393 modules, we focused on those that varied in relation to (1) the ethylene level-regulated time course and (2) 1-MCP treatment, using eigen patterns analyzed visually. The CG_0161 module, associated with ethylene levels, consists of 513 members, including ethylene and 21 volatiles such as ethyl esters, methyl esters, and ethanol (Table S5). This indicates that the module includes genes important for ethyl ester metabolism. TEA revealed that only IPR003676 (small auxin-up RNA; SAUR) and GO:0009733 (response to auxin) genes were over-represented according to ethylene levels, suggesting that changes in ethylene regulation may influence the crosstalk between ethylene and auxin. When the members of the CG_0161 module were further explored, five ethylene-related and 13 auxin-related Arabidopsis homologs were identified using TAIR (Table S6). Among the ethylene-related genes, the ones with the highest expression levels were homologs of Arabidopsis ACS8 (MD15G1302200, *MdACS1*), EIN3 (MD07G1053800, *MdEIN3*), and ACS12 (MD01G1186700, *MdACS12*). Among the 13 auxin-related genes in CG_0161, the highest expression was observed in the TIR1 gene (MD05G1325800). Additionally, among the ten genes annotated as SAUR homologs, eight were arranged in tandem repeats on chromosome 10, with the highest expression seen in MD10G1059600 (Table S6). CG_0161 also includes nine additional genes associated with phytohormones (Table S6).

### 3.4 Regulation of ester synthesis

#### 3.4.1 Ethyl ester regulation and ethanol pathway

**Fig. 6:**
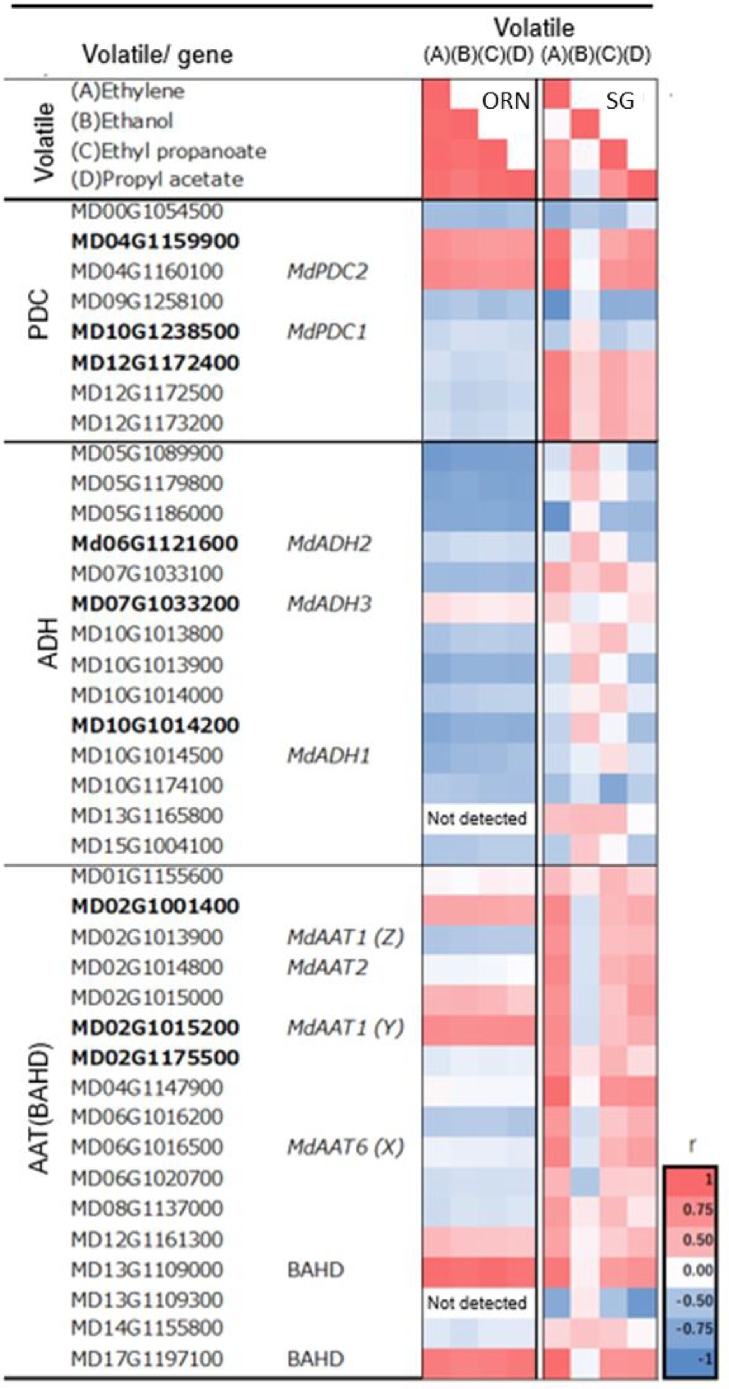
Correlation among ethylene production, volatile compounds, and gene expression across cultivars. The top three genes in the category, based on the maximum expression of each gene, are shown in bold. Volatiles (A)Ethylene, (B)Ethanol, (C)Ethyl propanoate, (D)Propyl acetate, PDC: pyrvate decarboxylase, ADH: alcohol dehydrogenase, AAT: alcohol acyltransferase, (Z) annotated by Larsen et al., 2019, (Y) annotated by Li, Yan, et al., 2023, (X) annotated by Yang et al., 2023, r: correlation coefficient.

As the sequential conversion of pyruvate to acetaldehyde to ethanol contributes to ethyl ester formation via the activity of PDC, ADH, and AAT enzymes, a gene search was conducted using the GDDH13 v1.1 genome. Additionally, previously reported genes with these enzymatic functions were also analyzed. In this study, genes with expression levels of 10 TPM or higher were selected for further analysis (Table S7). Fig. 6 illustrates the correlations between transcriptome and ethylene and its related compounds, including ethanol, ethyl esters (ethyl propanoate), and acetate esters (propyl acetate).

**Fig. 7:**
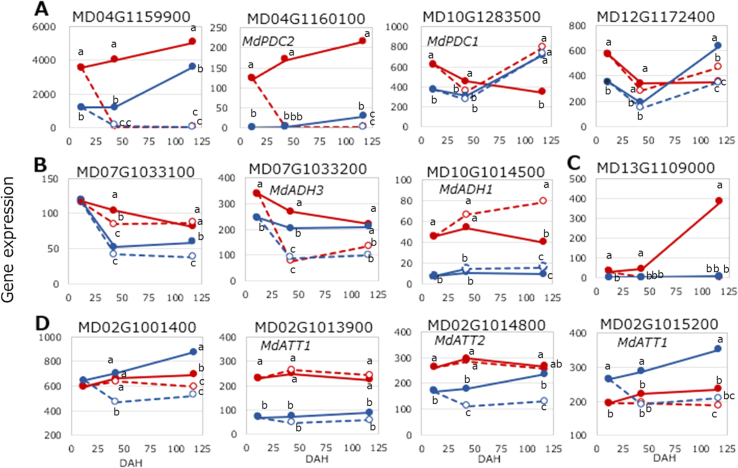
Expression of representative genes. A: PDC, B: ADH, C: BAHD, D: BAHD (AAT). Symbols are the same as in Fig. 2. Values with different letters differ significantly at the 5 % level within each DAH; Turkey's HSD test.

The initial step of the pathway involves PDC, which converts pyruvate into acetaldehyde. Among the 11 PDC genes listed in GDDH13 v1.1, eight were expressed at levels above 10 TPM. The previously reported PDC genes were included among these 11. MD04G1159900 and *MdPDC2* showed ethylene-dependent expression fluctuations (Fig. 2D, Fig. 6, Fig. 7A; Table S7). In MD04G1159900, expression was more than 20 times higher than in MD04G1160100 and was higher in ORN than in SG at CON conditions. Expression levels were significantly reduced by 1-MCP treatment in both cultivars. As shown in Fig. 6, MD04G1159900 and *MdPDC2* exhibited strong correlations with both ethylene and ethanol levels.

In the second step, from acetaldehyde to ethanol, a total of 21 ADH genes were annotated in GDDH13 v1.1 (Table S7). In addition to these, three genes previously reported as *MdADH1*–*MdADH3* were examined (Feng et al., 2021; Yang et al., 2016). Of these, 14 expressed genes were evaluated for their correlation with ethylene production and ethanol accumulation (Fig. 6, Fig. 7B; Table S7). As a result, a strong correlation was observed between ethylene production and ethanol, yet the majority of ADH genes did not exhibit a positive response to both ethylene and ethanol.

In the third step of the ethanol pathway, esterification is regulated by AAT. A review of the literature identified 32 putative AAT genes in apple (Li, Yan, et al., 2023; Yang et al., 2013; Yang et al., 2016; Yang et al., 2023). In the present study, nine of these genes were found to be expressed at TPM > 10. Among them, the following genes have been validated for their esterification ability: *MdAAT1* (MD02G1013900*,* Larsen et al., 2019), *MdAAT1* (MD02G1015200, Li, Yan, et al., 2023), *MdAAT2* (MD02G1014800), and *MdAAT6* (MD06G1020700, Yang et al., 2023). The JGI v1.1 genome includes 141 genes belonging to the BAHD acyltransferase family (HXXXD-type acyltransferases) (Li, Yan, et al., 2023), whereas 110 were detected in GDDH13 v1.1 in this study. Of these, 16 genes were found to be expressed at TPM > 10. The nine known AAT genes mentioned above with TPM > 10 were included in the BAHD family, except MD02G1001400. Thus, a total of 17 genes were selected for detailed analysis (Fig. 6, Fig. 7C, D; Tables S6). In ORN, the gene showing the highest similarity to esters, including ethyl and acetate esters, was MD13G1109000, which also exhibited the highest fold change (FC) and belonged to the CG_0161 module, the same cluster that included ethylene and ethyl esters in the WGCNA (Fig. 6, Table S5). The next most highly correlated gene was MD17G1197100, whose expression was slightly above TPM = 10 at its peak. Both were highly associated with ethyl and acetate esters (Fig. 6). Among the three known *MdAAT* genes located on chromosome 2, *MdAAT1* (MD02G1013900) and *MdAAT2* were more highly expressed in ORN (Fig. 7C); however, their expression did not significantly respond to ethylene (Fig. 6). *MdAAT1* (MD02G1015200) was positively correlated with ethylene in both cultivars and was consistently higher in SG.

## 4 Discussion

### 4.1 Cultivar-specific metabolite profiles and senescence in relation to ethylene and ethylene-related genes

General fruit quality parameters—such as sugar content, acidity, and firmness, as well as metabolites and ethylene levels, were compared between the cultivars. ORN exhibited higher pH values and began softening at 117 DAH. In addition, the levels of methyl and ethyl esters, ethanol, and sorbitol followed similar trends, increasing over time and surpassing those observed in SG. Notably, the increasing patterns of methyl and ethyl esters, methanol, and ethanol were suppressed by 1-MCP treatment along with ethylene. These components clustered into the same CG module as ethylene in the WGCNA analysis, suggesting a strong relationship between these compounds and ethylene biosynthesis. Ethyl esters have been reported are known to contribute to sweet aroma characteristics (Tanaka et al., 2020). The abundant ethyl esters in ORN may be central to its distinctive sweet flavor (Brown & Maloney, 2002; Yang et al., 2021) and could be associated with its higher ethylene production. In contrast, acetate esters (excluding methyl and ethyl types), γ-lactones, and mono- and sesquiterpenes (including terpene alcohols) were present in higher amounts in SG (Fig. 3), with large loading values along the PC2 axis (Fig. 4B), indicating these as characteristic aroma components of SG. Taken together, these findings suggest that differences in aroma profiles and senescence behavior between the two cultivars are largely driven by variation in ethylene production. However, in SG, ethanol and ethyl esters were very low even after ethylene levels increased. This implies that the downstream metabolic pathway from ethylene to ethanol and ethyl ester formation, and its regulatory mechanism, may differ fundamentally between the cultivars.

One of the key genes involved in ethylene biosynthesis during fruit ripening is *MdACS1* (Costa et al., 2005; Yang et al., 2013). Previous studies have shown that ORN possesses the ethylene-producing ACS1–1 allele, whereas SG is homozygous for the low ethylene-producing ACS1–2 allele (Kikuchi et al., 2017). The patterns of ethylene production and *MdACS1* expression observed in this study align with the ACS allele composition of each cultivar.

WGCNA analysis further revealed that auxin-related genes, including *MdTIR1* and ten SAUR genes, clustered with ethylene, *MdACS1,* and ethyl esters within the same co-expression (CG) module*.* This finding suggests a possible interaction between auxin and ethylene pathways, highlighting the need for further investigation into their crosstalk and roles in ester biosynthesis.

### 4.2 Regulation of ethyl ester synthesis

#### 4.2.1 Each step of the ethanol pathway

Ethanol, **a** common substrate **for** ethyl esters, **plays** a **pivotal role** in ethyl ester synthesis. The ethanol pathway **diverges** from the glycolysis–TCA cycle at a **key intermediate**, pyruvate. **In the first s**tep, **pyruvate is converted to** acetaldehyde, catalyzed by PDC. **The** subsequent oxidation of acetaldehyde to ethanol is **mediated** by ADH, while the **final** condensation of ethanol **with a** fatty acid is catalyzed by AAT.

To date, for the conversion of pyruvate to acetaldehyde, *MdPDC1* (MD10G1283500) and/or *MdPDC2* have been identified as critical genes for ethanol and ethyl ester synthesis under hypoxic conditions, depending on the cultivar (Li et al., 2022). However, in this study, the expression patterns of *MdPDC1* did not correspond to those of ethanol and ethyl esters in either cultivar. In contrast, MD04G1159900 and *MdPDC2* exhibited ethylene-dependent expression fluctuations. Taken together, these findings suggest that the conversion of pyruvate to acetaldehyde is predominantly regulated by MD04G1159900 and/or *MdPDC2*.

In the second step, the conversion of acetaldehyde to ethanol, considering the presence or absence of 1-MCP treatment and differences among cultivars, ethyl esters were found to be significantly more abundant in CON than in MCP and in ORN than in SG. Consequently, only *MdADH3* (MD07G1033200*)* satisfied the specified criteria of being higher in ORN and CON than in SG and MCP. However, when analyzed within cultivars, *MdADH3* did not show a positive correlation with ethylene and ethanol. This is because the expression of *MdADH3* decreased despite the increase in ethylene production over time. This partially agrees with Wang et al. (2018), who found that *MdADH3* expression was inhibited by 1-MCP treatment in ORN, although the FC was not as large as that observed for PDC at 23 days after treatment, and with Yang et al. (2016), who reported that *MdADH3* expression was upregulated by ethylene treatment in ‘Golden Delicious.’ However, in this study, even in ORN, which produced significant ethyl esters, no ADH gene showed expression patterns positively correlating with ethylene, PDC, and/or ethanol. In SG, all ADH genes showed negative correlations between ethylene and ethanol. Considering the pivotal role of ADH in ethanol synthesis driven by ethylene signaling, the following hypotheses are postulated for ORN: (1) *MdADH3* may be involved in the catalysis of acetaldehyde to ethanol synthesis, a process slightly stimulated by ethylene, though the negative feedback from the products may have contributed to downregulation of the gene during prolonged storage; and (2) the decarboxylation of pyruvate may be more rate-limiting than the alcohol dehydrogenation of acetaldehyde (Feng et al., 2021; Park et al., 2022; Yang et al., 2016). In SG, although *MdADH3* expression was equivalent to 75 % of that in ORN at DAH43, ethanol synthesis was significantly reduced (Fig. 3, Fig. 7B). This further supports the hypothesis that functional differences between ORN and SG in PDC, ADH, and/or their transcriptional regulation may exist between ORN and SG factors. However, given the absence of acetaldehyde data in this study, it was not possible to determine whether PDC or ADH exhibited impaired functionality.

Considering AAT gene expression and the substrate specificity of the esterification process, *MdAAT1* (MD02G1015200) may be involved in acetate ester synthesis, while *MdAAT1* (MD02G1013900) or *MdAAT2* may contribute to ethyl ester synthesis, as their expression was higher in the ethyl ester-rich ORN than in the acetate ester-rich SG. However, their expression levels were equivalent between CON and MCP, despite the several hundredfold increase in ethyl ester intensity from MCP to CON. If *MdAAT1* (MD02G1013900) or *MdAAT2* functioned as the enzymes catalyzing ethyl ester condensation, the process would not be rate-limiting, suggesting that the ethanol pool size may be the limiting factor.

The BAHD acyltransferase family is known for its diverse functions. In this study, the sequences of MD13G1109000 and MD17G1197100 were examined due to their strong correlation with ethylene, ethanol, and ethyl esters in ORN. However, their sequences significantly diverged from known AAT genes (data not shown), leaving their roles in ester synthesis unresolved.

#### 4.2.2 The key process for ethyl ester synthesis in ORN

An overview of the three steps of the ethanol pathway in Section 4.2.1 suggests that MD04G1159900 and *MdPDC2* contribute to the first step, *MdADH3* to the second step, and *MdAAT1* (MD02G1013900) and/or *MdAAT2* to the final step in ethyl ester synthesis in ORN. Correlation and gene expression analyses indicate that PDC genes MD04G1159900 and/or *MdPDC2* are critical for ethyl ester synthesis due to their role in driving the metabolite flow into the ethanol pathway. Furthermore, a strong association was observed between ethyl ester and ethanol levels, whereas no such response was detected in the expression of *MdADH3, MdAAT1* (MD02G1013900), and *MdAAT2*. The question of whether AAT enzyme activity or precursor availability is the rate-limiting factor in ester formation has been long debated (Defilippi et al., 2005; Souleyre et al., 2014; Yang et al., 2016). In the case of ethyl ester synthesis, precursor ethanol availability clearly emerged as the dominant factor in the current study.

#### 4.2.3 Suppressed ethyl ester synthesis in SG

In SG, ethanol synthesis was significantly reduced, even in the presence of ethylene at 33 μL hr^−1^ kg^−1^ in SG2C—a level higher than the ethylene production in ORN1C (Fig.2D, 3A). The observed ethanol levels in SG were lower than expected based on ethylene production levels in ORN. This finding suggests a possible inhibition of PDC or ADH function, which could lead to reduced ethanol levels—and consequently, lower ethyl ester production —in SG. More specifically, the inhibition appears to occur between PDC transcription and ethanol synthesis, as PDC gene expression was positively correlated with ethylene production. However, the exact point of inhibition remains unclear due to the absence of acetaldehyde data. If PDC function were intact but ADH activity impaired, acetaldehyde would be expected to accumulate, potentially leading to a distinctive sweaty odor—yet this was not observed. Therefore, it is more likely that PDC activity is compromised after transcription, resulting in reduced ethanol production. To clarify the underlying the inhibition of ethanol synthesis in SG, further studies are planned, including enzymatic assays and acetaldehyde quantification.

The sensory appeal of ethyl esters depends on various factors, including their concentration, associated texture characteristics, and the profile of accompanying metabolites. Notably, ethyl esters in apples following long-term storage may be linked to fruit senescence (Zhao et al., 2024). The naturally low ethyl ester synthesis observed in SG may thus be a desirable trait for breeding high-quality apple cultivars that retain a fresh-apple flavor even after extended storage.

### 4.3 Ethyl ester synthesis at hypoxia and at ripening/senescence

The synthesis of ethyl esters in apples has been reported to occur when the fruit is subjected to low-oxygen conditions. Two PDC genes, *MdPDC1* and *MdPDC2*, have been identified as the primary contributors to this process. Li et al. (2022) reported that *MdPDC1* and *MdPDC2* were upregulated by controlled atmosphere (CA) treatment and were associated with higher CO_2_ tolerance in ‘Golden Delicious’. Park et al. (2022) observed that both *MdPDC1* and *MdPDC2* were upregulated in ‘Empire’ and ‘Jonagold’ whereas ‘Fuji’ upregulated only *MdPDC2*. In watercored apples, a high abundance of ethyl esters has also been reported (Tanaka et al., 2020; Wada et al., 2021). Notably, higher expression of *MdPDC1* was observed in the watercored region compared to the unaffected region and non-watercored ‘Fuji’ (Wada et al., 2021). In the present study, we used ORN and SG, cultivars resistant to watercore, to clearly distinguish ethylene-driven from watercore-driven ethyl ester synthesis. The experiment was conducted under normoxic conditions, and results demonstrated an association of MD04G1159900 and *MdPDC2* with ethylene production during ripening, while *MdPDC1* remained unresponsive in both ORN and SG. This finding is consistent with the report by Yang et al. (2016), which showed that *MdPDC2* was induced during fruit ripening, whereas *MdPDC1* expression remained unaffected in ‘Golden Delicious.’ Similarly, the results align with those of Wang et al. (2018), who reported that *MdPDC2* expression was induced during ripening and reduced by 1-MCP treatment in ORN. Consequently, we postulate that the initial step of ethyl ester synthesis driven by ethylene is predominantly regulated by MD04G1159900 and/or *MdPDC2*. *MdPDC2* may play a role in ethyl ester synthesis induced by both hypoxia and elevated ethylene, whereas MD04G1159900 appears to respond solely to elevated ethylene. Notably, MD04G1159900 is located in tandem with *MdPDC2* and consistently exhibits higher expression levels; FC in expression relative to *MdPDC2* within the same sample ranged from 20 to 150, with similar fluctuation patterns. Moreover, MD04G1159900 was expressed continuously, even when ethylene levels were below 1 μL h^−1^ kg^−1^. To the best of our knowledge, this gene has only been reported by Li et al. (2022). Further studies are needed to elucidate the specific functions of these PDC genes. The cultivar-specific relationships between ethyl esters, acetate esters, alcohols, and the genes encoding PDC, ADH, and AAT enzymes are visualized in a network diagram in Fig. S1 for reference.

### 4.4 Overview of pyruvate metabolism

**Fig. 8:**
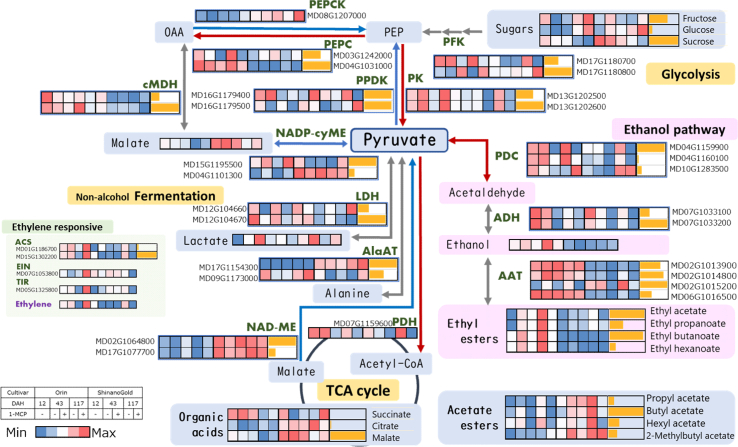
Overview of pyruvate-related metabolism during ripening/senescence in ‘Orin’ and ‘Shinano Gold’. Color charts show relative intensities of gene expression and metabolite levels. Bar graphs to the right indicate the relative maximum expression for each gene or metabolite within the group. Red and blue arrows indicate processes enhanced or inhibited by ethylene, respectively. **Abbreviations.** PFK, Phosphofructokinase; PK, pyruvate kinase; PPDK, Pyruvate phosphate dikinase; PEPC, phosphoenolpyruvate carboxylase; PEPCK, phosphoenolpyruvate carboxylase kinase; PDC, pyruvate decarboxylase; ADH, alcohol dehydrogenase; AAT, alcohol acyltransferase; LDH, lactate dehydrogenase; AlaAT, alanine aminotransfelase; PDH, Pyruvate dehydrogenase; cMDH, cytosol malate dehydrogenase; NAD-ME, NAD-malic enzyme; NADP-cyME; NADP-cytosol malic enzyme; ACS, 1-amino-cyclopropane-1-carboxylate synthase; EIN, ethylene-insensitive; TIR, transport inhibitor response; PEP, phosphoenolpyruvate; OAA, oxaloacetic acid. (For interpretation of the references to color in this figure legend, the reader is referred to the web version of this article.)

An extended overview of the impact of ethylene on glycolysis and the subsequent transition to the TCA cycle was analyzed (Fig. 8). Red and blue arrows indicate processes that were upregulated or downregulated by ethylene, respectively, including those with high or low ratios in ORN compared to SG. It is widely accepted that under low-oxygen conditions, pyruvate can initially be converted into either lactate or ethanol via lactate dehydrogenase (LDH) and PDC, respectively. In a subsequent stage under progressive hypoxia, pyruvate is converted to alanine by alanine aminotransferase (AlaAT) (Boeckx et al., 2019). An increase in alanine has been observed in ‘Braeburn’ apples after long-term storage under high CO_2_ conditions (Hatoum et al., 2014) and in the watercored tissue of ‘Fuji’ and ‘Koutoku’ apples (Tanaka et al., 2016; Tanaka et al., 2020). Another pyruvate metabolic pathway—the conversion of pyruvate to citrate by pyruvate dehydrogenase (PDH)-is known to be inhibited under hypoxic conditions due to the accumulation of NADH. However, in the present study, neither LDH nor AlaAT genes were induced by elevated ethylene, as might be expected under hypoxic conditions. Moreover, the carbon flow into the TCA cycle via PDH was upregulated with increasing ethylene levels. This finding aligns with the absence of an increase in acetoin or acetol—compounds typically elevated under hypoxic condition (Table S2). This suggests that the activated ethanol pathway results in a distinct flavor profile depending on whether it is induced by low oxygen or by ripening/senescence, despite the shared presence of ethyl esters. The perceived palatability of these changes is worth exploring, especially in relation to the interaction between ethyl esters and other flavor components in apple.

Hypoxia is thought to lead to pyruvate accumulation through the activation of glycolysis and inhibition of PDH activity (Boeckx et al., 2019; Plaxton & Podestá, 2006). Although our study was conducted under aerobic conditions, we estimate that elevated ethylene also stimulated pyruvate synthesis during the ripening process of ORN (Fig. 8). These activations of the ethanol pathway may act as regulatory mechanisms for maintaining proper pyruvate levels under both aerobic and hypoxic conditions. Similarly, ethylene treatment of persimmon fruit has been reported to activate PDC and ADH, resulting in increased acetaldehyde and ethanol levels (Min et al., 2012). This ethylene-driven activation of the ethanol pathway may represent a common mechanism across a wide range of fruits.

Genes involved in the ethanol pathway have been shown to exhibit substantial functional variation depending on the cultivar (Park et al., 2022). This genetic diversity likely plays a significant role in shaping ester composition among different apple cultivars. The diversity of PDC, ADH, and AAT genes and their impact on flavor profiles beyond ORN and SG cultivars is currently under investigation.

### 4.5 Acetate esters and other volatiles

Acetate esters were distributed across multiple modules in the WGCNA, suggesting that the regulation of their synthesis is likely multifaceted. This contrasts with ethyl esters, which are closely associated with ethanol and ethylene within the same module. It is estimated that acetate ester synthesis involves complex metabolic processes, including the LOX pathway, β-oxidation, malate degradation via malic enzyme, and esterification, each of which is likely regulated independently. In this study, ethylene production was found to be positively correlated with acetate ester levels within individual cultivars. However, the low ethylene-producing cultivar SG exhibited higher acetate ester levels than the high ethylene-producing cultivar ORN. This variation may be attributed to differing effects of ethylene on the various processes involved in acetate ester synthesis. Additionally, the mechanism underlying acetate ester accumulation in SG requires further investigation.

## 5 Conclusion

This study revealed that ORN produced significant amounts of ethyl esters under aerobic conditions, with production levels proportional to ethylene synthesis. In contrast, SG produced very low levels of ethyl esters, even when ethylene was present at high concentrations. In ORN, PDC plays an important role in ethylene-driven ethyl ester synthesis, specifically through MD04G1159900 and *MdPDC2* (MD04G11601000). This finding indicates that ethanol availability is more critical than AAT activity for the rate of ethyl ester synthesis. In contrast, an inhibitory mechanism appears to exist in SG because the level of ethyl ester synthesis was much lower than expected based on its ethylene production. This suggests that the function of PDC and/or ADH in the ethanol synthesis pathway is impaired in SG, likely at a post-transcriptional level following PDC expression. The molecular basis of this reduced ethyl ester productivity in SG warrants further investigation. This experiment suggests a potential diversity in genes associated with the ethanol pathway among cultivars. To regulate ester profiles, including ethyl esters, a comparative study of the sequence and function of PDC, ADH, AAT, and their transcription factor genes across a wide range of cultivars is necessary. Understanding the regulatory mechanisms governing ester synthesis will contribute to developing improved apple storage techniques and breeding more flavorful cultivars.

## CRediT authorship contribution statement

**Fukuyo Tanaka:** Writing – review & editing, Writing – original draft, Investigation, Funding acquisition, Formal analysis, Data curation, Conceptualization. **Ryoichi Yano:** Writing – review & editing, Writing – original draft, Software, Formal analysis, Data curation. **Keiki Okazaki:** Writing – review & editing, Investigation, Formal analysis. **Hikari Kuchikata:** Writing – review & editing, Formal analysis. **Jimpachi Masuda:** Writing – review & editing, Formal analysis. **Satoshi Kasai:** Writing – review & editing, Resources. **Miho Tatsuki:** Writing – review & editing, Writing – original draft, Investigation, Funding acquisition.

## Declaration of competing interest

The authors declare the following financial interests/personal relationships which may be considered as potential competing interests: Fukuyo Tanaka reports financial support was provided by Japan Society for the Promotion of Science. Miho Tatsuki reports financial support was provided by Japan Society for the Promotion of Science. If there are other authors, they declare that they have no known competing financial interests or personal relationships that could have appeared to influence the work reported in this paper.

## Data Availability

Supporting data for the study's findings are available in the article's supplementary material and in the National Center for Biotechnology Information (NCBI) under the accession number PRJNA1255739

## Appendix A Supplementary data

Supplementary material 1 Image 1

Supplementary material 2 Image 2

## References

[bib389] Abouelenein D., Acquaticci L., Alessandroni L., Borsetta G., Caprioli G., Mannozzi C., Mustafa A.M. (2023). Volatile Profile of Strawberry Fruits and Influence of Different Drying Methods on Their Aroma and Flavor: A Review. Molecules.

[bb0005] Aprea E., Charles M., Endrizzi I., Laura Corollaro M., Betta E., Biasioli F., Gasperi F. (2017). Sweet taste in apple: The role of sorbitol, individual sugars, organic acids and volatile compounds. Scientific Reports.

[bb0015] Arabidopsis Genome Initiative (2000). Analysis of the genome sequence of the flowering plant *Arabidopsis thaliana*. Nature.

[bb0020] Argenta L.C., Mattheis J.P., Fan X., Finger F.L. (2004). Production of volatile compounds by Fuji apples following exposure to high CO_2_ or low O_2_. Journal of Agricultural and Food Chemistry.

[bb0025] Asikin Y., Shimoda K., Takeuchi M., Maekawa R., Kamiyoshihara Y., Takara K., Wada K. (2022). Free and glycosidically bound volatile compounds in Okinawan pineapple (*Ananas comosus*). Applied Sciences.

[bb0030] Blankenship S.M., Dole J.M. (2003). 1-Methylcyclopropene: A review. Postharvest Biology and Technology.

[bb0035] Boeckx J., Pols S., Hertog M.L.A.T.M., Nicolaï B.M. (2019). Regulation of the central carbon metabolism in apple fruit exposed to postharvest low-oxygen stress. Frontiers in Plant Science.

[bb0040] Bolger A.M., Lohse M., Usadel B. (2014). Trimmomatic: A flexible trimmer for Illumina sequence data. Bioinformatics.

[bb0045] Brown S., Maloney K. (2002). Apple cultivars: A Geneva perspective. New York Fruit Quarterly.

[bb0050] Cliff M., Stanich K., Trujillo J.M., Toivonen P., Forney C.F. (2011). Determination and prediction of odor thresholds for odor active volatiles in a neutral apple juice matrix. Journal of Food Quality.

[bb0055] Costa F., Stella S., Van de Weg W.E., Guerra W., Cecchinel M., Dallavia J., Sansavini S. (2005). Role of the genes md-ACO1 and md-ACS1 in ethylene production and shelf life of apple (*Malus domestic*a **Borkh**). Euphytica.

[bb0060] Daccord N., Celton J.-M., Linsmith G., Becker C., Choisne N., Schijlen E., Bucher E. (2017). High-quality de novo assembly of the apple genome and methylome dynamics of early fruit development. Nature Genetics.

[bb0065] Defilippi B.G., Kader A.A., Dandekar A.M. (2005). Apple aroma: Alcohol acyltransferase, a rate limiting step for ester biosynthesis, is regulated by ethylene. Plant Science.

[bb0070] Dixon J., Hewett Errol W. (2000). Exposure to hypoxia conditions alters volatile concentrations of apple cultivars. Journal of the Science of Food and Agriculture.

[bb0075] Fan X., Mattheis J.P. (2001). 1-Methylcyclopropene and storage temperature influence responses of ‘Gala’apple fruit to gamma irradiation. Postharvest Biology and Technology.

[bb0080] Fan X., Mattheis J.P., Buchanan D. (1998). Continuous requirement of ethylene for apple fruit volatile synthesis. Journal of Agricultural and Food Chemistry.

[bb0090] Fellman J.K., Rudell D.R., Mattinson D.S., Mattheis J.P. (2003). Relationship of harvest maturity to flavor regeneration after CA storage of ‘delicious’ apples. Postharvest Biology and Technology.

[bb0095] Feng S., Yan C., Zhang T., Ji M., Tao R., Gao H. (2021). Comparative study of volatile compounds and expression of related genes in fruit from two apple cultivars during different developmental stages. Molecules.

[bb0100] Fuhrmann E., Grosch W. (2002). Character impact odorants of the apple cultivars Elstar and cox Orange. Food/Nahrung.

[bb0105] Hatoum D., Annaratone C., Hertog M.L.A.T.M., Geeraerd A.H., Nicolai B.M. (2014). Targeted metabolomics study of ‘Braeburn’ apples during long-term storage. Postharvest Biology and Technology.

[bb0115] Holland D., Larkov O., Bar-Ya’akov I., Bar E., Zax A., Brandeis E., Lewinsohn E. (2005). Developmental and varietal differences in volatile ester formation and acetyl-CoA: Alcohol acetyl transferase activities in apple (Malus domestica Borkh.) fruit. Journal of Agricultural and Food Chemistry.

[bb0120] Honda C., Tanaka F., Ohmori Y., Tanaka A., Komazaki K., Izumi K., A. (2024). Differences in the aroma profiles of seedless-treated and nontreated “shine Muscat” grape berries decrease with ripening. The Horticulture Journal.

[bb0125] Horiuchi N., Moriguchi S., Fukuda H., Ichimura N., Kato Y., Baba Y. (1986). Composition of volatile compounds of apple fruits in relation to cultivars. Journal of the Japanese Society for Horticultural Science.

[bb0130] Igarashi M., Hatsuyama Y., Harada T., Fukasawa-Akada T. (2016). Biotechnology and apple breeding in Japan. Breeding Science.

[bb0135] Kanno Y., Hirata M., Mitani K., Shirota S., Onodera T., Toko K. (2021). Visualization of flavor of sake using taste sensor and gas chromatography-mass spectrometry. Electronics and Communications in Japan.

[bb0140] Kikuchi T., Kasajima I., Morita M., Yoshikawa N. (2017). Practical DNA markers to estimate apple (*Malus× domestica***Borkh**.) skin color, ethylene production and pathogen resistance. Journal of Horticulture.

[bb0145] Kim D., Paggi J.M., Park C., Bennett C., Salzberg S.L. (2019). Graph-based genome alignment and genotyping with HISAT2 and HISAT-genotype. Nature Biotechnology.

[bb0150] Komthong P., Katoh T., Igura N., Shimoda M. (2006). Changes in the odours of apple juice during enzymatic browning. Food Quality and Preference.

[bb0160] Langfelder P., Horvath S. (2008). WGCNA: An R package for weighted correlation network analysis. BMC Bioinformatics.

[bb0165] Larsen B., Migicovsky Z., Jeppesen A.A., Gardner K.M., Toldam-Andersen T.B., Myles S., Pedersen C. (2019). Genome-wide association studies in apple reveal loci for aroma volatiles, sugar composition, and harvest date. The Plant Genome.

[bb0170] Li G., Jia H., Li J., Li H., Teng Y. (2016). Effects of 1-MCP on volatile production and transcription of ester biosynthesis related genes under cold storage in ‘Ruanerli’ pear fruit (*Pyrus ussuriensis* maxim). Postharvest Biology and Technology.

[bb0175] Li R., Yan D., Tan C., Li C., Song M., Zhao Q., Liu C. (2023). Transcriptome and metabolomics integrated analysis reveals *MdMYB94* associated with esters biosynthesis in apple (*Malus*× Domestica). Journal of Agricultural and Food Chemistry.

[bb0180] Li X., Wang J., Su M., Zhang M., Hu Y., Du J., Ye Z. (2023). Multiple-statistical genome-wide association analysis and genomic prediction of fruit aroma and agronomic traits in peaches. Horticulture Research.

[bb0185] Li Y., Zheng C., Wang C., Golding J.B., Ru L. (2022). Comparative transcriptome reveals molecular mechanism in apple genotypes differing in CO_2_ tolerance in CA storage. Postharvest Biology and Technology.

[bb0190] Liu G., Bi J., Chen Q. (2025). Impact of postharvest ripening on peach quality: Aroma release and formation mechanism. Food Chemistry.

[bb0195] Liu Z., Liang T., Kang C. (2023). Molecular bases of strawberry fruit quality traits: Advances, challenges, and opportunities. Plant Physiology.

[bb0200] Mehinagic E., Royer G., Symoneaux R., Jourjon F., Prost C. (2006). Characterization of odor-active volatiles in apples: Influence of cultivars and maturity stage. Journal of Agricultural and Food Chemistry.

[bb0205] Mimura N., Isogai A., Iwashita K., Bamba T., Fukusaki E. (2014). Gas chromatography/mass spectrometry based component profiling and quality prediction for Japanese sake. Journal of Bioscience and Bioengineering.

[bib386] Min T.Y., Yin X.R., Shi Y.N., Luo Z.R., Yao Y.C., Grierson D., Chen (2012). Ethylene-responsive transcription factors interact with promoters of ADH and PDC involved in persimmon (Diospyros kaki) fruit de-astringency. Journal of Experimental Botany.

[bb0210] Munoz-Gonzalez C., Perez-Jimenez M., Criado C., Pozo-Bayon M.A. (2019). Effects of ethanol concentration on oral aroma release after wine consumption. Molecules.

[bb0215] Niu Y., Wang R., Xiao Z., Zhu J., Sun X., Wang P. (2019). Characterization of ester odorants of apple juice by gas chromatography-olfactometry, quantitative measurements, odour threshold, aroma intensity and electronic nose. Food Research International.

[bb0220] Okazaki K., Tanahashi T., Kato Y., Suzuki I., Tanaka F., Ohwaki Y. (2020). Metabolic indices related to leaf marginal necrosis associated with potassium deficiency in tomato using GC/MS metabolite profiling. Journal of Bioscience and Bioengineering.

[bb0225] Park D., Shoffe Y.A., Algul B.E., Watkins C.B. (2022). Fermentative metabolism of three apple cultivars during storage under low partial pressures of oxygen. Postharvest Biology and Technology.

[bb0230] Pertea M., Pertea G.M., Antonescu C.M., Chang T.-C., Mendell J.T., Salzberg S.L. (2015). StringTie enables improved reconstruction of a transcriptome from RNA-seq reads. Nature Biotechnology.

[bb0235] Plaxton W.C., Podestá F.E. (2006). The functional organization and control of plant respiration. Critical Reviews in Plant Sciences.

[bb0240] R Core Team (2021). R: A language and environment for statistical computing. https://www.R-project.org/.

[bb0245] Roberts G., Spadafora N.D. (2020). Analysis of apple flavours: The use of volatile organic compounds to address cultivar differences and the correlation between consumer appreciation and aroma profiling. Journal of Food Quality.

[bb0250] Rowan D., Hunt M., Dimouro A., Alspach P., Weskett R., Volz R., Chagne D. (2009). Profiling fruit volatiles in the progeny of a ‘Royal Gala’ x ‘granny smith’ apple (Malus x domestica) cross. Journal of Agricultural and Food Chemistry.

[bb0255] Shi F., Zhou X., Zhou Q., Tan Z., Yao M.-M., Wei B.-D., Ji S.-J. (2018). Membrane lipid metabolism changes and aroma ester loss in low-temperature stored Nanguo pears. Food Chemistry.

[bb0260] Soomro T., Jordan M., Watts S., Migicovsky Z., Forney C.F., Song J., Myles S. (2023). Genomic insights into apple aroma diversity. Fruit Research.

[bb0265] Souleyre E.J.F., Chagné D., Chen X., Tomes S., Turner R.M., Wang M.Y., Atkinson R.G. (2014). The *AAT1* locus is critical for the biosynthesis of esters contributing to ripe apple flavour in ‘Royal Gala’ and ‘granny smith’ apples. The Plant Journal.

[bb0270] Styger G., Prior B., Bauer F.F. (2011). Wine flavor and aroma. Journal of Industrial Microbiology and Biotechnology.

[bb0280] Tanaka F., Hayakawa F., Tatsuki M. (2020). Flavor and texture characteristics of ‘Fuji’ and related apple (*Malus domestica* L.) cultivars, focusing on the rich watercore. Molecules.

[bb0285] Tanaka F., Miyazawa T., Okazaki K., Tatsuki M., Ito T. (2015). Sensory and metabolic profiles of “Fuji” apples (*Malus domestica***Borkh**) grown without synthetic agrochemicals: The role of ethylene production. Bioscience, Biotechnology, and Biochemistry.

[bb0290] Tanaka F., Okazaki K., Kashimura T., Ohwaki Y., Tatsuki M., Sawada A., Miyazawa T. (2016). Profiles and physiological mechanisms of sensory attributes and flavor components in watercored apple (in Japanese). Journal of the Japanese Society for Food Science and Technology.

[bb0295] Tatsuki M., Endo A., Ohkawa H. (2007). Influence of time from harvest to 1-MCP treatment on apple fruit quality and expression of genes for ethylene biosynthesis enzymes and ethylene receptors. Postharvest Biology and Technology.

[bb0310] Tokitomo Y., Steinhaus M., Buttner A., Schieberle P. (2005). Odor-active constituents in fresh pineapple (*Ananas comosus* [L.] Merr.) by quantitative and sensory evaluation. Bioscience, Biotechnology, and Biochemistry.

[bb0315] Ueda Y., Tsuda A., Bai J.-H., Fujishita N., Chachin K. (1992). Characteristic pattern of aroma ester formation from banana, melon, and strawberry with reference to the substrate specificity of ester synthetase and alcohol contents in pulp. Nippon Shokuhin Kogyo Gakkaishi.

[bb0320] Van Gemert L. (2003).

[bb0325] Wada H., Nakata K., Nonami H., Erra-Balsells R., Tatsuki M., Hatakeyama Y., Tanaka F. (2021). Direct evidence for dynamics of cell heterogeneity in watercored apples: Turgor-associated metabolic modifications and within-fruit water potential gradient unveiled by single-cell analyses. Horticulture Research.

[bb0330] Wan C.Y., Wilkins T.A. (1994). A modified hot borate method significantly enhances the yield of high-quality RNA from cotton (*Gossypium hirsutum* L.). Analytical Biochemistry.

[bb0335] Wang A., Yamakake J., Kudo H., Wakasa Y., Hatsuyama Y., Igarashi M., Harada T. (2009). Null mutation of the MdACS3 gene, coding for a ripening-specific 1-aminocyclopropane-1-carboxylate synthase, leads to long shelf life in apple fruit. Plant Physiology.

[bb0340] Wang S., Saito T., Ohkawa K., Ohara H., Suktawee S., Ikeura H., Kondo S. (2018). Abscisic acid is involved in aromatic ester biosynthesis related with ethylene in green apples. Journal of Plant Physiology.

[bb0345] Watkins C.B., Nock J.F., Whitaker B.D. (2000). Responses of early, mid and late season apple cultivars to postharvest application of 1-methylcyclopropene (1-MCP) under air and controlled atmosphere storage conditions. Postharvest Biology and Technology.

[bib388] Yang S., Meng Z., Li Y., Chen R., Yang Y., Zhao Z. (2021). Evaluation of physiological characteristics, soluble sugars, organic acids and volatile compounds in ‘Orin’apples (Malus domestica) at different ripening stages. Molecules.

[bb0350] Yang S., Yu J., Yang H., Zhao Z. (2023). Genetic analysis and QTL mapping of aroma volatile compounds in the apple progeny Fuji’ × ‘Cripps pink’. Frontiers in Plant Science.

[bb0355] Yang X., Song J., Campbell-Palmer L., Fillmore S., Zhang Z. (2013). Effect of ethylene and 1-MCP on expression of genes involved in ethylene biosynthesis and perception during ripening of apple fruit. Postharvest Biology and Technology.

[bb0360] Yang X., Song J., Du L., Forney C., Campbell-Palmer L., Fillmore S., Zhang Z. (2016). Ethylene and 1-MCP regulate major volatile biosynthetic pathways in apple fruit. Food Chemistry.

[bb0365] Yano R., Ariizumi T., Nonaka S., Kawazu Y., Zhong S., Mueller L., Ezura H. (2020). Comparative genomics of muskmelon reveals a potential role for retrotransposons in the modification of gene expression. Communications Biology.

[bb0370] Yano R., Li F., Hiraga S., Takeshima R., Kobayashi M., Toda K., Ishimoto M. (2025). The genomic landscape of gene-level structural variations in Japanese and global soybean *Glycine max* cultivars. Nature Genetics.

[bb0375] Young J.C., Chu C.L.G., Lu X., Zhu H. (2004). Ester variability in apple varieties as determined by solid-phase microextraction and gas chromatography−mass spectrometry. Journal of Agricultural and Food Chemistry.

[bb0380] Zhao H., Zhang S., Ma D., Liu Z., Qi P., Wang Z., Wang X. (2024). Review of fruits flavor deterioration in postharvest storage: Odorants, formation mechanism and quality control. Food Research International.

[bb0385] Zhao J., Liu J., Wang F., Wang S., Feng H., Xie X., Fang C. (2020). Volatile constituents and ellagic acid formation in strawberry fruits of selected cultivars. Food Research International.

