# Supplementary material for: Regulation of ethyl ester synthesis in two apple (Malus domestica) cultivars: Insights from integrated metabolomic and transcriptomic analyses
Source: Food Chem (Oxf). 2025 Aug 5;11:100282. doi: 10.1016/j.fochms.2025.100282 (PMC12357301; doi:10.1016/j.fochms.2025.100282)
Supplement: Supplementary file 1 — Supplementary material 1 [file mmc1.pptx]

## Slide 1
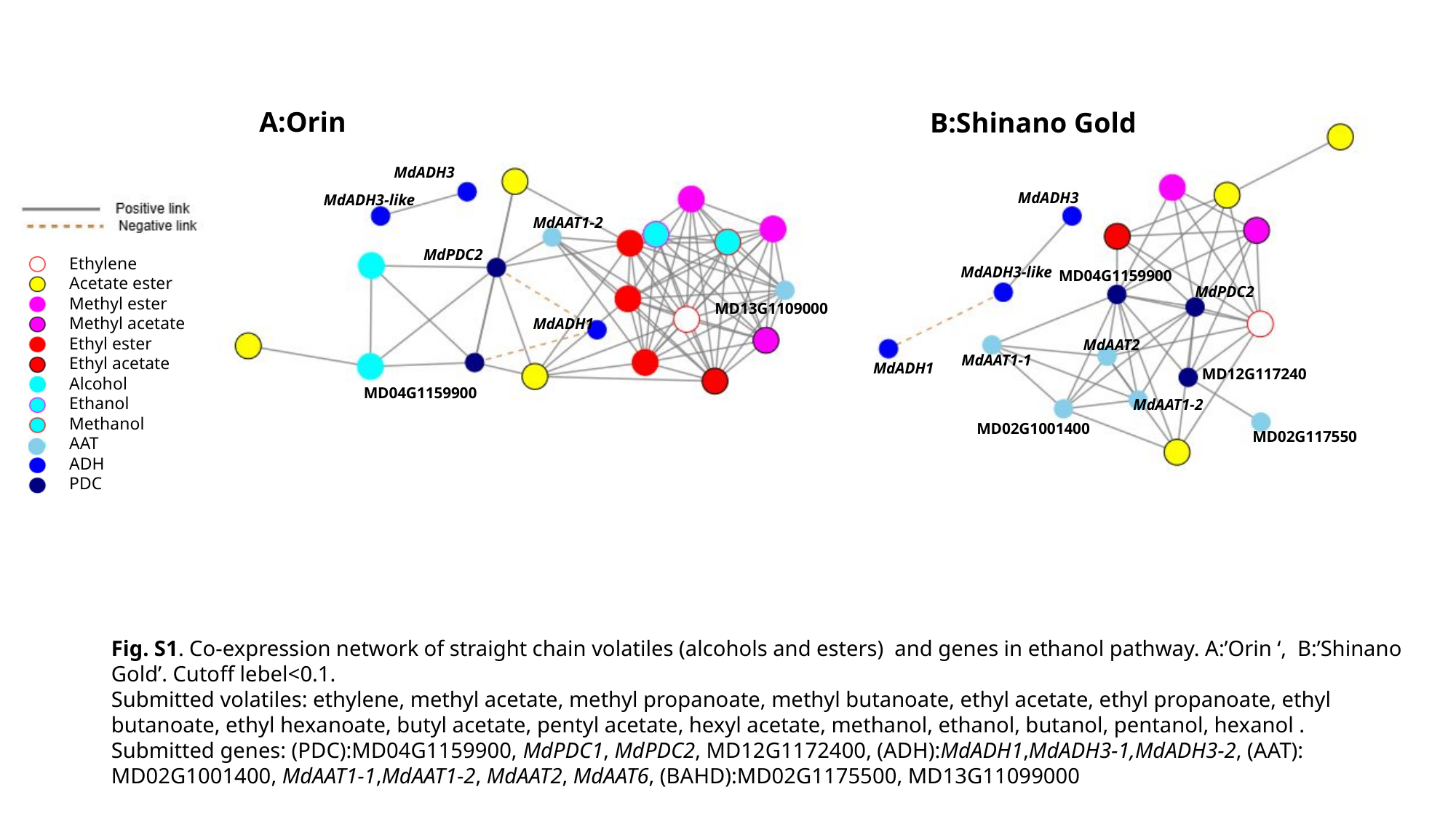

A:Orin
B:Shinano Gold
MdADH3
MdADH3
MdADH3-like
MdAAT1-2
MdPDC2
Ethylene
Acetate ester
Methyl ester
Methyl acetate
Ethyl ester
Ethyl acetate
Alcohol
Ethanol
Methanol
AAT
ADH
PDC
MdADH3-like
MD04G1159900
MdPDC2
MD13G1109000
MdADH1
MdAAT2
MdAAT1-1
MdADH1
MD12G117240
MD04G1159900
MdAAT1-2
MD02G1001400
MD02G117550
Fig. S1. Co-expression network of straight chain volatiles (alcohols and esters) and genes in ethanol pathway. A:’Orin ‘, B:’Shinano Gold’. Cutoff lebel<0.1.
Submitted volatiles: ethylene, methyl acetate, methyl propanoate, methyl butanoate, ethyl acetate, ethyl propanoate, ethyl butanoate, ethyl hexanoate, butyl acetate, pentyl acetate, hexyl acetate, methanol, ethanol, butanol, pentanol, hexanol .
Submitted genes: (PDC):MD04G1159900, MdPDC1, MdPDC2, MD12G1172400, (ADH):MdADH1,MdADH3-1,MdADH3-2, (AAT): MD02G1001400, MdAAT1-1,MdAAT1-2, MdAAT2, MdAAT6, (BAHD):MD02G1175500, MD13G11099000
